# Direct versus indirect measures of mixed emotions in predictive models: a comparison of predictive validity, multicollinearity, and the influence of confounding variables

**DOI:** 10.3389/fpsyg.2023.1231845

**Published:** 2023-08-21

**Authors:** Vincent Y. S. Oh

**Affiliations:** Singapore University of Social Sciences, School of Humanities and Behavioural Sciences, Singapore, Singapore

**Keywords:** mixed emotions, direct measures, minimum index, indirect measures, multicollinearity, emotional ambivalence, emotional complexity, well-being

## Abstract

Mixed emotions have been assessed using both direct measures that utilize self-report questionnaires as well as indirect measures that are computed from scores of positive and negative emotions. This study provides a pre-registered methodological examination on the use of direct and indirect measures of mixed emotions in predictive models. Two samples (*N* = 749) were collected, and path analyses were performed to compare direct measures and indirect measures in predicting psychological conflict, receptivity, and well-being, controlling for demographics, positive emotions, and negative emotions. We also tested whether trait dialecticism, need for cognition, social desirability, or acquiescence could account for these associations. In both samples, results suggest that indirect measures may be more susceptible to multicollinearity when controlling for positive and negative emotions. Specifically, variance inflation factors (VIF) were consistently higher for indirect measures calculated using the minimum index (MIN; VIF_Sample-1_ = 3.53; VIF_Sample-2_ = 9.46) than direct measures (VIF_Sample-1_ = 2.52; VIF_Sample-2_ = 1.68). Direct measures remained consistently associated with increased conflict and reduced coherence upon controlling for positive and negative emotions, while indirect measures remained consistently associated only with increased conflict. We found little evidence that response biases explained associations between direct measures or indirect measures with each of the outcomes. Specifically, associations between mixed emotions with psychological conflict, receptivity, and well-being largely remained unchanged in models that controlled for trait dialecticism, need for cognition, social desirability, or acquiescence. Implications and recommendations based on our findings are discussed.

## Introduction

There is growing interest in studying mixed emotions in which positive and negative emotions co-occur (Larsen and McGraw, 2011; Oh and Tong, 2022), and two general approaches have been used to measure mixed emotions. The first approach involves direct measures which directly instruct participants to report experiences of mixed emotions on self-report questionnaires (Barford and Smillie, 2016). The second approach involves indirect measures which do not directly ask participants about mixed emotions, but instead calculate them based on scores for positive and negative emotions; most commonly, the minimum index (MIN) has been recommended as a valid indirect measure which is computed by taking the smaller of two scores between positive and negative emotions (Larsen et al., 2017). There is substantial variability even within these approaches. For example, different items are often included in different direct measures (Barford and Smillie, 2016; Oh and Tong, 2021), and indirect measures can be calculated either from single scores for positivity and negativity (Berrios et al., 2018) or could be calculated from averaged scores for positivity and negativity (Oh, 2022). Nevertheless, evidence suggests that both direct measures and indirect measures computed using MIN validly assess mixed emotions when they occur (Berrios et al., 2015).

However, little has been done to address statistical issues related to their use in predictive models testing associations between mixed emotions and key outcomes. Specifically, the extent to which direct measures versus indirect measures predict key outcomes the same way, the extent to which these measures may be affected by statistical issues such as multicollinearity, and the extent to which these measures may be confounded by response biases remains unclear. We performed a pre-registered exploratory study addressing these issues.

Based on meta-analytic evidence assessing the measurement of mixed emotions following experimental manipulations, some potential limitations with indirect measures as computed using MIN have been suggested by Berrios et al. (2015). For example, as MIN works by taking the smaller score between positive and negative emotions, it may provide lower-boundary estimates of mixed emotions, which may lead to some degree of range restriction. Additionally, effect sizes for detecting mixed emotions were also found to be smaller when using indirect measures. Preliminary evidence also suggests that indirect measures may be more susceptible to multicollinearity when entered alongside positive and negative emotions (Oh, 2022)—in particular, as most people experience more positive affect than negative affect in daily life, the smaller of two scores between the two will commonly be the score for negative affect. Hence, scores for MIN are often very strongly correlated with negative affect, leading to multicollinearity. The typical consequence of multicollinearity is that errors may be inflated and statistical power may be reduced, making it more difficult to detect genuine effects and leading to Type II errors (Mason and Perreault, 1991). Moreover, some researchers have argued that multicollinearity could also be linked to Type I errors (Kalnins, 2022).

If so, the overall consequence of multicollinearity is that it may introduce interpretive difficulties. A common recommendation for dealing with multicollinearity is to remove multicollinear variables from regression equations—however, this is less feasible in correlational work aiming to establish a unique explanatory role of mixed emotions. In experimental designs, multicollinearity is less critical as such designs allow researchers to separate mixed emotions, positive emotions, and negative emotions into their respective conditions and analyses conducted generally do not require linear regressions (e.g., Rees et al., 2013; Oh and Tong, 2021). In correlational contexts, however, ruling out the explanatory role of positive and negative emotions virtually necessitates their statistical control to conclude that any associations are independently explainable by mixed emotions (Berrios et al., 2018; Dejonckheere et al., 2019; Oh and Tong, 2022). An empirical comparison of direct and indirect measures in predictive models is crucial in providing researchers with empirical guidance about the use of these measures.

An additional concern is that various response biases may limit the utility of either direct or indirect measures, or both. Firstly, compared to measures of single emotions (e.g., “happy”), direct measures of mixed emotions involve more complex items describing multiple emotions (e.g., “both happy and sad,” “a mixture of two opposing emotions”), and individuals high on trait cognitive complexity may be more likely to endorse these items. Secondly, as both negative emotions and mixed emotions are often uncomfortable states that are seen as undesirable (Rothman et al., 2017), social desirability may reduce the likelihood of reporting such states (Oh and Tong, 2021), leading to underestimated scores on both direct measures and MIN. Thirdly, high scores on self-reported measures could reflect acquiescence, the tendency to agree to self-report items haphazardly (Kam, 2016). To the extent that acquiescence inflates self-reported scores for positive emotions, negative emotions, as well as mixed emotions, both direct measures and MIN could be biased towards artificially inflated scores. It is thus crucial to provide empirical evidence that associations between mixed emotions and later outcomes are not attributable to such biases, which would lend weight to existing evidence about mixed emotions and support the use of these measures in future research.

We performed a pre-registered exploratory investigation of these methodological issues. We collected two separate samples which differ only in the emotion measures administered—as different researchers often use different scales, this allows replication of our conclusions across different measures of emotions. We assessed key outcomes based on existing evidence on mixed emotions. Given that mixed emotions fundamentally involve the co-occurrence of opposing affective states, several perspectives suggest that conflict is the central hallmark of mixed emotions (van Harreveld et al., 2009; Vaccaro et al., 2020), and we hence assessed psychological conflict as a key outcome variable. Some evidence also suggests that mixed emotions serve integrative functions, such that they signal the need to process a complex environment *via* enhancing receptivity to conflicting perspectives or new behaviors (Rees et al., 2013; Oh and Tong, 2021), and we hence also assessed psychological receptivity. Finally, diverse evidence has linked mixed emotions to well-being outcomes, most commonly conceptualized using eudaimonic measures. For example, Oh (2022) found that mixed emotions in daily life were linked to poorer eudaimonic well-being, while Berrios et al. (2018) and Oh and Tong (2021) instead found that mixed emotions in more specific contexts such as a graduation ceremony or the COVID-19 pandemic were linked positively to eudaimonic well-being. As such, we also assessed eudaimonic well-being using a four-dimension scale adapted from Costin and Vignoles (2020). We performed path analyses comparing the predictive validity of direct versus indirect measures of mixed emotions for these outcomes, with and without controls for positive and negative emotions. Additional analyses were performed to determine whether trait dialecticism, need for cognition, social desirability, and acquiescence could confound associations between measures of mixed emotions and these outcomes.

## Method

### Participants

This study is pre-registered at https://aspredicted.org/3CQ_DVK, with a follow-up pre-registration at https://aspredicted.org/1TS_MV5. Two separate samples were collected—the procedures are identical for both samples with the exception of the emotion measures administered. Participants from the United States with at least a 99% approval rate and at least 5,000 HITs completed were recruited *via* Amazon MTurk and were reimbursed USD$1.50 for their participation. Based on evidence that correlations stabilize at sample sizes above 250 (Schönbrodt and Perugini, 2013), we sought to collect at least 300 participants in each sample following exclusions. Sample 1 included 414 participants, while Sample 2 included 433 participants. After excluding participants who failed attention checks, reported being distracted, or exceeded the 97.5 percentile on off-task behavior (Permut et al., 2019), 369 participants were included in Sample 1, while 380 participants were included in Sample 2 (demographic details provided in Table 1). Data and *R* codes are available at https://osf.io/8z45f/?view_only=0ef3fe4c38034891a5e54bc784021053.

**Table 1:** Descriptive statistics of all variables in both samples.

	Sample 1			Sample 2		
	*M (α)*	SD	Range	*M (α)*	SD	Range
Age	44.05	13.34	22 to 83	45.17	13.47	21 to 89
Gender	0.41	0.49	151 Male, 214 Female	0.43	0.50	163 Male, 216 Female
Education	8.23	2.00	2 to 12	8.35	2.04	3 to 12
Income	6.32	2.99	2 to 14	6.51	3.36	1 to 14
Positive Emotions	3.57 (0.96)	1.42	1 to 7	4.18 (0.92)	1.39	1 to 7
Negative Emotions	1.79 (0.96)	1.08	1 to 6.5	1.67 (0.93)	1.00	1 to 5.6
Mixed Emotions (Direct Measure)	2.02 (0.97)	1.14	1 to 5.78	2.30 (0.94)	1.21	1 to 6.31
Mixed Emotions (MIN)	1.61	0.85	1 to 5.42	1.59	0.87	1 to 5.4
Conflict	2.48 (0.93)	1.46	1 to 7	2.55 (0.93)	1.48	1 to 7
Receptivity	4.86 (0.85)	1.24	1 to 7	5.12 (0.84)	1.12	1 to 7
Meaning	5.47 (0.89)	1.58	1 to 7	5.44 (0.92)	1.61	1 to 7
Coherence	5.21 (0.77)	1.33	1 to 7	5.20 (0.77)	1.32	1 to 7
Purpose	5.41 (0.84)	1.49	1 to 7	5.35 (0.83)	1.45	1 to 7
Mattering	4.81 (0.87)	1.76	1 to 7	4.82 (0.87)	1.79	1 to 7
Trait Dialecticism	3.58 (0.80)	0.82	1 to 5.85	3.57 (0.80)	0.86	1 to 5.77
Need for Cognition	4.60 (0.94)	1.71	1 to 7	4.62 (0.94)	1.67	1 to 7
Social Desirability	4.56 (0.88)	1.13	1 to 7	4.55 (0.88)	1.12	1.12 to 7
Acquiescence	4.23 (0.27)	0.54	2.75 to 6.25	4.18 (0.29)	0.54	2.75 to 6.38

### Measures

Descriptive statistics and internal consistency statistics are presented in Table 1. Items for the respective scales were averaged following reverse-coding of negatively-worded items. Items for all measures are provided in Appendix A. All items were rated on 7-point Likert scales.

#### Emotions

Participants were instructed to indicate how much they felt various state emotions during the past 15 min. All items were administered on 7-point Likert scales (1 = *Did not feel the emotion at all*, 4 = *Felt the emotion moderately*, 7 = *Felt the emotion very much*).

##### Sample 1

Emotion measures from Oh (2022) were directly adapted. Specifically, 23 items measured mixed emotions (*α* = 0.97); 19 items measured positive emotions (*α* = 0.96); and 18 items measured negative emotions (*α* = 0.96). MIN was calculated by taking the smaller of the two scores between positive emotions and negative emotions.

##### Sample 2

Mixed emotions were measured using 13 items (*α* = 0.94) adapted from Barford and Smillie (2016), while positive (*α* = 0.92) and negative (*α* = 0.93) emotions were, respectively, measured using 10 items from the Positive and Negative Affect Schedule (PANAS; Watson et al., 1988). MIN was calculated by taking the smaller of the two scores between positive emotions and negative emotions.

#### Outcome variables

Three key outcome variables were assessed. All items were administered on 7-point Likert scales (1 = *Not at all*; 4 = *Somewhat*; 7 = *Very much*). *Psychological conflict* was assessed using a 7-item scale (*α_Sample-1_* = 0.93; *α_Sample-2_* = 0.93) assessing current psychological contradiction and conflict. *Receptivity* was assessed using a 7-item scale (*α_Sample-1_* = 0.85; *α_Sample-2_* = 0.84) assessing current willingness to consider conflicting information and perspectives and try new behaviors (Rees et al., 2013; Oh and Tong, 2021). *Well-being* was measured using a 16-item meaning in life scale adapted from Costin and Vignoles (2020)—the scale comprises four separate dimensions which were analyzed separately: *meaning* (*α_Sample-1_* = 0.89; *α_Sample-2_* = 0.92), *coherence* (*α_Sample-1_* = 0.77; *α_Sample-2_* = 0.77), *purpose* (*α_Sample-1_* = 0.84; *α_Sample-2_* = 0.83), *mattering* (*α_Sample-1_* = 0.87; *α_Sample-2_* = 0.87).

#### Covariates

Age, gender, education level, and income were assessed as demographic covariates. *Trait dialecticism* was measured using 13 items (*α_Sample-1_* = 0.80; *α_Sample-2_* = 0.80) from Spencer-Rodgers et al. (2015); *need for cognition* was measured using 6 items (*α_Sample-1_* = 0.94; *α_Sample-2_* = 0.94) from Lins de Holanda Coelho et al. (2020); *social desirability* was measured using 16 items (*α_Sample-1_* = 0.88; *α_Sample-2_* = 0.88) from Hart et al. (2015); and *acquiescence* was measured using a nonsense scale comprising 16 items (*α_Sample-1_* = 0.27; *α_Sample-2_* = 0.29) which are of diverse content and include both positively-worded and negatively-worded items (Kam, 2016). Scores on the acquiescence scale are hence meaningless and purely reflect acquiescence biases towards self-reported items, which is also reflected in the low internal consistency statistics. All items were administered on 7-point Likert scales. Items for trait dialecticism and need for cognition used the following anchors (1 = *Not at all*; 4 = *Somewhat*; 7 = *Very much*); while items for social desirability and acquiescence used the following anchors (1 = *Strongly Disagree*; 7 = *Strongly Agree*).

#### Exclusion criteria

Participants who failed an attention check (“I always obey laws, but for this question select “3″ to show that you are paying attention.”) or reported being distracted (1 = *distracted*, 0 = *not distracted*) were excluded. Additionally, a qualitative attention check was administered at the end of the study in which participants were instructed to write 3 sentences describing a happy memory—participants who did not comply with these instructions were excluded.

## Results

Correlation matrices for each sample are reported in Supplementary Tables S1, S2, while the full analytic models with all covariates are reported in Supplementary Tables S3–S6. Direct measures were strongly correlated with MIN (*r_Sample-1_* = 0.81; *r_Sample-2_* = 0.66; *p*s < 0.001), suggesting substantial convergence in the constructs they assess. Furthermore, as shown in Table 2, path analyses adjusting only for demographic variability indicate that direct measures and MIN were related to the outcome variables in overwhelmingly similar ways. Specifically, both measures predicted increased psychological conflict, reduced meaning, coherence, and purpose, but not receptivity. The sole difference is that MIN predicted reduced mattering in Sample 2, while direct measures did not.

**Table 2:** Results for direct measures and indirect measures (MIN) of mixed emotions on all key outcome variables in each sample.

	Sample 1				Sample 2			
	Direct measure		MIN		Direct measure		MIN	
DV	*β*	*p*	*β*	*p*	*β*	*p*	*β*	*p*
Controlling for demographics (age, gender, education, and income)								
Conflict	0.65^***^	<0.001	0.55^***^	<0.001	0.65^***^	<0.001	0.56^***^	<0.001
Receptivity	<0.001	0.99	−0.05	0.34	0.01	0.92	−0.06	0.27
Meaning	−0.21^***^	<0.001	−0.27^***^	<0.001	−0.10^*^	0.049	−0.18^***^	<0.001
Coherence	−0.35^***^	<0.001	−0.35^***^	<0.001	−0.21^***^	<0.001	−0.24^***^	<0.001
Purpose	−0.27^***^	<0.001	−0.32^***^	<0.001	−0.16^**^	0.001	−0.20^***^	<0.001
Mattering	−0.04	0.42	−0.10	0.060	−0.09	0.074	−0.16^**^	0.002
Controlling for demographics, positive emotions, and negative emotions								
Conflict	0.65^***^	<0.001	0.38^***^	<0.001	0.57^***^	<0.001	0.41^**^	0.001
Receptivity	0.04	0.59	−0.02	0.80	0.002	0.97	−0.06	0.71
Meaning	0.01	0.86	0.04	0.61	−0.08	0.14	0.05	0.72
Coherence	−0.26^***^	<0.001	−0.15	0.055	−0.19^***^	<0.001	0.001	1.00
Purpose	−0.18^**^	0.008	−0.23^**^	0.005	−0.17^**^	0.002	−0.02	0.86
Mattering	0.05	0.45	0.04	0.61	−0.10	0.073	−0.12	0.38
Controlling for demographics, positive emotions, negative emotions, and trait dialecticism								
Conflict	0.56^***^	<0.001	0.31^***^	<0.001	0.53^***^	<0.001	0.39^**^	0.002
Receptivity	−0.16^*^	0.037	−0.13	0.12	−0.07	0.29	−0.08	0.59
Meaning	0.04	0.56	0.05	0.48	−0.05	0.42	0.06	0.64
Coherence	−0.22^**^	0.001	−0.11	0.14	−0.16^**^	0.005	0.02	0.91
Purpose	−0.15^*^	0.045	−0.20^*^	0.013	−0.11^*^	0.037	−0.004	0.98
Mattering	0.06	0.39	0.04	0.59	−0.07	0.21	−0.11	0.42
Controlling for demographics, positive emotions, negative emotions, and need for cognition								
Conflict	0.65^***^	<0.001	0.38^***^	<0.001	0.57^***^	<0.001	0.41^**^	0.001
Receptivity	0.02	0.75	−0.04	0.66	−0.05	0.38	−0.10	0.47
Meaning	0.01	0.92	0.03	0.65	−0.10	0.065	0.03	0.80
Coherence	−0.26^***^	<0.001	−0.15^*^	0.050	−0.21^***^	<0.001	−0.01	0.93
Purpose	−0.19^**^	0.006	−0.23^**^	0.004	−0.19^**^	0.001	−0.04	0.77
Mattering	0.05	0.46	0.04	0.63	−0.12^*^	0.032	−0.14	0.32
Controlling for demographics, positive emotions, negative emotions, and social desirability								
Conflict	0.62^***^	<0.001	0.35^***^	<0.001	0.55^***^	<0.001	0.40^**^	0.001
Receptivity	0.05	0.50	−0.02	0.87	−0.002	0.97	−0.06	0.71
Meaning	0.06	0.35	0.08	0.28	−0.06	0.29	0.05	0.72
Coherence	−0.20^**^	0.001	−0.09	0.24	−0.17^**^	0.001	<0.001	1.00
Purpose	−0.13^*^	0.049	−0.18^*^	0.022	−0.14^**^	0.010	−0.02	0.85
Mattering	0.09	0.17	0.08	0.33	−0.08	0.19	−0.12	0.37
Controlling for demographics, positive emotions, negative emotions, and acquiescence								
Conflict	0.65^***^	<0.001	0.38^***^	<0.001	0.57^***^	<0.001	0.40^**^	0.002
Receptivity	0.05	0.55	−0.01	0.91	0.01	0.89	−0.05	0.76
Meaning	0.01	0.83	0.05	0.54	−0.06	0.30	0.09	0.47
Coherence	−0.26^***^	<0.001	−0.15	0.056	−0.19^***^	0.001	0.01	0.93
Purpose	−0.18^**^	0.008	−0.23^**^	0.005	−0.14^**^	0.008	0.02	0.87
Mattering	0.06	0.40	0.06	0.49	−0.07	0.20	−0.07	0.61
Controlling for all covariates								
Conflict	0.55^***^	<0.001	0.30^***^	<0.001	0.52^***^	<0.001	0.37^**^	0.003
Receptivity	−0.14^*^	0.045	−0.11	0.17	−0.13^*^	0.034	−0.12	0.38
Meaning	0.06	0.31	0.09	0.23	−0.02	0.70	0.09	0.48
Coherence	−0.18^**^	0.005	−0.07	0.33	−0.16^**^	0.005	0.01	0.95
Purpose	−0.12	0.088	−0.18^*^	0.027	−0.09	0.081	0.02	0.85
Mattering	0.09	0.19	0.09	0.27	−0.04	0.46	−0.07	0.57

^*^p < 0.05, ^**^p < 0.01, ^***^p < 0.001.

We next examined path models in which positive emotions and negative emotions are controlled. In both samples, direct measures were correlated with positive (*r_Sample-1_* = 0.28; *r_Sample-2_* = 0.17; *p*s < 0.001) and negative emotions (*r_Sample-1_* = 0.71; *r_Sample-2_* = 0.60; *p*s < 0.001). In Sample 1, MIN was also correlated with positive (*r_Sample-1_* = 0.25, *p* < 0.001) and negative emotions (*r_Sample-1_* = 0.80; *p* < 0.001). However, in Sample 2, MIN was not correlated with positive emotions (*r_Sample-2_* = 0.03, *p* = 0.56) and was very strongly correlated with negative emotions (*r_Sample-2_* = 0.94, *p* < 0.001).

Multicollinearity is typically indexed using variance inflation factors (VIF) or tolerance statistics, which assess the degree to which variances of regression coefficients are inflated due to collinearity (O’brien, 2007). As VIFs and tolerance statistics are functionally and mathematically similar, we report only VIFs. Examining multicollinearity statistics when entering mixed emotions alongside positive and negative emotions, direct measures had lower multicollinearity scores (VIF_Sample-1_ = 2.52; VIF_Sample-2_ = 1.68) compared to MIN (VIF_Sample-1_ = 3.53; VIF_Sample-2_ = 9.46). MIN suffered from particularly severe multicollinearity in Sample 2, which made further analysis inadvisable—we nevertheless analyzed models using MIN to illustrate the impact of multicollinearity in predictive models where mixed emotions are entered alongside positive and negative emotions. As shown in Table 2, controlling for positive and negative emotions generally attenuates the predictive validity of mixed emotions. However, whereas direct measures remained predictive of conflict, coherence, and purpose in both samples, MIN remained consistently predictive only of conflict, and predicted purpose only in Sample 1 but not Sample 2. These findings are consistent with previous evidence suggesting that inflated multicollinearity reduces statistical power, making it harder to detect effects.

Finally, we examined models in which potential confounding factors were entered to determine whether the predictive validity of direct measures or MIN may be influenced by these factors. Both direct measures (*r_Sample-1_* = 0.33; *r_Sample-2_* = 0.32; *p*s < 0.001) and MIN (*r_Sample-1_* = 0.24; *r_Sample-2_* = 0.26; *p*s < 0.001) were correlated with trait dialecticism. Direct measures (*r_Sample-1_* = −0.02; *r_Sample-2_* = 0.07; *p*s > 0.15) and MIN (*r_Sample-1_* = −0.06; *r_Sample-2_* = −0.05; *p*s > 0.20) were not correlated with need for cognition. Both direct measures (*r_Sample-1_* = −0.28; *r_Sample-2_* = −0.25; *p*s < 0.001) and MIN (*r_Sample-1_* = −0.29; *r_Sample-2_* = −0.32; *p*s < 0.001) were negatively correlated with social desirability. Finally, in Sample 1, neither direct measures nor MIN were correlated with acquiescence (*r_Sample-1-DM_* = 0.04; *r_Sample-1-MIN_* = 0.07; *p*s > 0.15). However, in Sample 2, both direct measures and MIN were correlated with acquiescence (*r_Sample-2-DM_* = 0.24; *r_Sample-2-MIN_* = 0.18; *p*s < 0.001).

As shown in Table 2, associations between direct measures and MIN with the key outcomes remained relatively unchanged in models when these constructs were entered as covariates, suggesting that the key predictive validities of either direct measures and MIN are not attributable to response biases due to trait cognitive complexity, social desirability, or acquiescence. One exception is that in full models where all covariates are entered, the significant negative association between direct measures of mixed emotions with purpose fell to marginal significance in both samples. Additionally, controlling for trait dialecticism caused the non-significant association between direct measures and receptivity to turn significant in Sample 1, while controlling for need for cognition caused the non-significant association between direct measures and mattering to turn significant in Sample 2. In the full models with all covariates controlled, the non-significant association between direct measures and receptivity became significant in both samples. These are likely suppression effects resulting from statistical artifacts due to these covariates and should be interpreted cautiously.

Given that both direct measures and MIN significantly predicted psychological conflict, we further performed seemingly unrelated regressions^1^ to compare the standardized coefficients of direct measures and MIN for psychological conflict in the full models with all covariates included. In sample 1, direct measures were significantly more predictive of psychological conflict than MIN, χ^2^ (1) = 7.14, *p* = 0.008, though the difference was non-significant in Sample 2, χ^2^ (1) = 1.30, *p* = 0.25. Taken together with the finding that direct measures but not MIN predicted coherence in both samples, these findings provide partial support for the idea that direct measures may have greater predictive validity than MIN in models controlling for positive and negative emotions.

## Discussion

In two samples, direct and indirect measures were strongly positively correlated and were related to outcome variables in similar ways, which suggests that both measures are likely to be valid indicators of mixed emotions (Berrios et al., 2015; Larsen et al., 2017) and also suggests some convergence between objective and subjective indicators of ambivalence (Ng et al., 2022). However, consistent with Oh (2022), we found that indirect measures were more vulnerable to multicollinearity when entered alongside positive and negative emotions, which complicates their use in predictive models seeking to conclude an additive role of mixed emotions over positive and negative emotions (Dejonckheere et al., 2019). Whereas direct measures remained predictive of increased conflict and reduced coherence in both samples after controlling for positive and negative emotions, indirect measures remained consistently predictive only of conflict. Finally, in models accounting for trait dialecticism, need for cognition, social desirability, and acquiescence, the predictive validities of direct and indirect measures largely remained consistent, suggesting that response biases from these constructs are unlikely to account for findings based on these measures.

Issues with multicollinearity are complicated. Rules of thumbs for determining multicollinearity (e.g., VIF < 5) provide simple heuristics for determining the severity of multicollinearity, but are not definitive (O’brien, 2007). For example, previous work suggests that a major consequence of multicollinearity is reduced statistical power, and at larger sample sizes which are statistically more powerful or with larger effect sizes that are easier to detect, even relatively higher multicollinearity statistics may be less problematic (Mason and Perreault, 1991; O’brien, 2007). Our findings are consistent with this to some extent. For example, indirect measures remained predictive of psychological conflict despite relatively severe multicollinearity, likely because large effect sizes for this variable alleviate losses in statistical power. In contrast, for outcome variables where effect sizes are smaller, indirect measures are less able to detect effects due to losses of statistical power. Generally, though higher multicollinearity does not guarantee statistical issues, such difficulties become more likely as multicollinearity increases.

We caution against simplistically interpreting our findings as implying that direct measures are not susceptible to multicollinearity, or that indirect measures are unusable in predictive models—neither of these conclusions are warranted. At small sample sizes or when trying to detect small effect sizes, even relatively lower multicollinearity statistics such as those found when using direct measures could introduce interpretive difficulties (Mason and Perreault, 1991; O’brien, 2007). Indirect measures remain valid and reliable in predictive models, but may be more appropriate in analytic situations where reduced statistical power is not a major problem, such as if large effect sizes are expected or if large sample sizes are available. A further implication is that it may be prudent to plan for larger sample sizes in anticipation of losses in statistical power that may occur due to multicollinearity for studies that seek to include indirect measures of mixed emotions along with positive and negative emotions in regression models.

Some limitations should be noted. Firstly, our findings apply to the measurement of mixed emotions in daily life and may not generalize to other contexts. For example, in adversity contexts (e.g., Oh and Tong, 2021) where negative emotions may be reported at higher levels than positive emotions, MIN is less likely to track negative emotion scores and we may likely expect less multicollinearity between MIN and negative affect. Additionally, experimental contexts of mixed emotions induced momentarily and examinations of more specific variants of mixed emotions (e.g., Oh and Tong, 2022) may potentially yield different results than global examinations of mixed-valenced mood. Future examinations should thus also compare the statistical properties of direct and indirect measures in more specific situational contexts of measurement.

Secondly, we have focused on comparing direct measures and MIN on certain statistical issues, but other important considerations should be noted. For example, apart from statistical considerations, methodological considerations are pertinent. In studies that assess positive and negative emotions over longer periods, such as over several days or weeks, MIN is not appropriate as an index of simultaneous co-occurrence, and direct measures may be more suitable (Oh and Tong, 2022). Additionally, even within direct and indirect measures, there is substantial variability in their statistical properties depending on factors like item choice. For example, there was greater multicollinearity in the direct measures of Sample 1 compared to Sample 2, and there was also greater multicollinearity in the indirect measures of Sample 2 compared to Sample 1. Moreover, as indirect measures are computed from two separate scores, a potential limitation of indirect measures is that estimates based on indirect measures may be especially imprecise due to them being influenced by the error variances of both positive emotion scores and negative emotion scores. As MIN provides a lower-boundary estimate of mixed emotions (Berrios et al., 2015), range restriction could speculatively also result in some statistical issues with the use of MIN. Finally, though we have focused on MIN given its prevalence in the literature, we note that there are other forms of indirect measures that may potentially yield different results from MIN.^2^ These are important issues that remain to be addressed in future work.

Though our primary aim is methodological, we briefly discuss theoretical implications. Firstly, strong positive associations between mixed emotions and psychological conflict support existing perspectives that conflict is the central characteristic of mixed emotions (van Harreveld et al., 2009; Vaccaro et al., 2020). Secondly, our findings conceptually replicate evidence that naturalistic experiences of mixed emotions may interfere with eudaimonic indicators of well-being (Oh, 2022), though we found consistent associations primarily with coherence but not other components of well-being. This is also in line with the idea that mixed emotions fundamentally involve conflict, which suggests that any detrimental implications of mixed emotions could occur primarily on dimensions of well-being involving a sense of conflict or inconsistency, such as coherence. Some evidence has also linked mixed emotions negatively to hedonic measures of well-being such as life satisfaction (Sun et al., 2021), and further research is needed to identify other conditions under which mixed emotions may or may not interfere with well-being outcomes (e.g., Berrios et al., 2018). Thirdly, we did not replicate evidence linking mixed emotions to increased receptivity (Rees et al., 2013; Oh and Tong, 2021). One possibility is that mixed emotions in daily life may primarily be conflicting and may interfere with integrative effects like receptivity (Oh, 2022). Alternatively, receptivity effects could be limited to certain contexts. Oh and Tong (2021) examined mixed emotions arising from the COVID-19 pandemic and Rees et al. (2013) specifically examined the mixed emotion ‘happy-sad’, while the present study examines generalized mixed emotions from daily life. Future research should examine these possibilities.

Overall, we recommend transparently reporting both direct and indirect measures wherever appropriate to provide comprehensive evidence, especially in studies where the goal is in detecting the occurrence of mixed emotions (Berrios et al., 2015). In predictive models, our findings support these measures as being relatively unconfounded by biases due to trait cognitive complexity, social desirability, and acquiescence, though they also advise interpretive caution when performing regression analyses with indirect measures while controlling for positive and negative emotions.

## Data availability statement

The datasets presented in this study can be found in online repositories. The names of the repository/repositories and accession number(s) can be found in the article/Supplementary material.

## Ethics statement

The studies involving humans were approved by National University of Singapore Departmental Ethics Review Committee. The studies were conducted in accordance with the local legislation and institutional requirements. The participants provided their written informed consent to participate in this study.

## Author contributions

The author confirms being the sole contributor of this work and has approved it for publication.

## Conflict of interest

The author declares that the research was conducted in the absence of any commercial or financial relationships that could be construed as a potential conflict of interest.

## Publisher’s note

All claims expressed in this article are solely those of the authors and do not necessarily represent those of their affiliated organizations, or those of the publisher, the editors and the reviewers. Any product that may be evaluated in this article, or claim that may be made by its manufacturer, is not guaranteed or endorsed by the publisher.

## Footnotes

^1^This analysis was not initially pre-registered, but was performed at the suggestion of Reviewer 2. We are grateful for the suggestion.

^2^As suggested by Reviewer 2, another indirect approach is the use of standardized residuals (Grossmann et al., 2016; Larsen et al., 2017). However, given that a single-session cross-sectional design was employed, this approach may be less appropriate as it is more typical in within-subjects designs where participants provide emotions data across multiple measurement occasions, which then allows data across measurement occasions to be aggregated. Nevertheless, in the interest of transparency, we explored the use of standardized residuals as well and we reported these analyses in Supplementary Analyses A.
