# Supplementary material for: Direct versus indirect measures of mixed emotions in predictive models: a comparison of predictive validity, multicollinearity, and the influence of confounding variables
Source: Front Psychol. 2023 Aug 21;14:1231845. doi: 10.3389/fpsyg.2023.1231845 (PMC10475543; doi:10.3389/fpsyg.2023.1231845)
Supplement: Supplementary file 1 [file Data_Sheet_1.docx]

**Supplementary Analyses A**

Larsen et al. (2017) also suggested the use of residualized MIN as another possible indirect approach to measuring mixed emotions while also accounting for mean-level positive and negative emotions. However, this has been used primarily in within-subjects methods where participants provide multiple instances of emotion reports across time points or situations, and aggregates were then calculated (e.g., Grossmann et al., 2016). As the methodology of the existing samples involves a single-session cross-sectional study, this approach may be less appropriate. Nevertheless, in the interest of transparency and exploration, we test and report this approach here.

We calculated standardized residuals in both samples by performing a linear regression predicting (1) direct measures of mixed emotions and (2) MIN using positive and negative emotions, followed by saving the standardized residual scores of each participant. For parsimony, we refer to these as residualized direct measures (RDM) and residualized MIN (RMIN) below. We first performed bivariate correlations between RDM and RMIN, with the key outcomes tested. The results are summarized below.

Bivariate correlations between residualized direct measures, residualized MIN, and all outcomes.

|  | Sample 1 | | | |  | Sample 2 | | | |
| --- | --- | --- | --- | --- | --- | --- | --- | --- | --- |
|  | RDM | | RMIN | |  | RDM | | RMIN | |
| DV | *r* | *p* | *r* | *p* |  | *r* | *p* | *r* | *p* |
| Conflict | .40^***^ | <.001 | .11^*^ | .034 |  | .43^***^ | <.001 | .002 | .96 |
| Receptivity | .02 | .67 | -.01 | .81 |  | .01 | .92 | -.01 | .84 |
| Meaning | .004 | .93 | .08 | .11 |  | -.06 | .21 | .04 | .46 |
| Coherence | -.15^**^ | .004 | -.002 | .96 |  | -.16^**^ | .002 | .04 | .40 |
| Purpose | -.13^*^ | .016 | -.07 | .19 |  | -.14^**^ | .008 | .02 | .69 |
| Mattering | .02 | .67 | .04 | .41 |  | -.08 | .10 | -.02 | .65 |

^*^ *p* < .05, ^**^ *p* < .01, ^***^ *p* < .001. RDM = Standardized Residuals of Positive and Negative Emotions Predicting Direct Measures of Mixed Emotions; RMIN = Standardized Residuals of Positive and Negative Emotions Predicting MIN of Mixed Emotions.

As shown in this table, in both samples, RDM was correlated with higher psychological conflict, lower coherence, and lower purpose in life, which is similar to the results reported in the main analyses using direct measures of mixed emotions. However, the results for RMIN showed little convergence with the main analyses. RMIN was weakly correlated with psychological conflict in Sample 1 and uncorrelated with psychological conflict in Sample 2, and was uncorrelated with all other outcome variables in both samples.

Next, using these standardized residuals, we then performed linear path analyses predicting the key outcomes, controlling for demographics (age, gender, education level, income) and all other covariates (trait dialecticism, need for cognition, social desirability, and acquiescence). The results are summarized below.

Path analyses predicting all outcomes using residualized direct measures (RDM) and residualized MIN (RMIN).

| Controlling for age, gender, education level, income, trait dialecticism, need for cognition, social desirability, and acquiescence | | | | | | | | | |
| --- | --- | --- | --- | --- | --- | --- | --- | --- | --- |
|  | Sample 1 | | | |  | Sample 2 | | | |
|  | RDM | | RMIN | |  | RDM | | RMIN | |
| DV | *β* | *p* | *β* | *p* |  | *β* | *p* | *β* | *p* |
| Conflict | .29^***^ | <.001 | .07 | .13 |  | .33^***^ | <.001 | .02 | .65 |
| Receptivity | -.06 | .16 | -.04 | .40 |  | -.09 | .069 | -.02 | .67 |
| Meaning | .10^*^ | .036 | .11^*^ | .016 |  | -.01 | .84 | .04 | .40 |
| Coherence | -.06 | .17 | .02 | .62 |  | -.10^*^ | .028 | .03 | .49 |
| Purpose | -.03 | .49 | -.04 | .34 |  | -.06 | .15 | .02 | .69 |
| Mattering | .10 | .052 | .08 | .11 |  | -.03 | .51 | -.02 | .62 |

^*^ *p* < .05, ^**^ *p* < .01, ^***^ *p* < .001. RDM = Standardized Residuals of Positive and Negative Emotions Predicting Direct Measures of Mixed Emotions; RMIN = Standardized Residuals of Positive and Negative Emotions Predicting MIN of Mixed Emotions.

Upon including covariates, RDM remained predictive of psychological conflict in both samples, but remained predictive of coherence only in Sample 2 and not Sample 1. A statistical artifact in which the non-significant bivariate association between RDM and meaning became significant also occurred, but only in Sample 1. Generally, the results using RDM partially replicated the main findings, though they are generally weaker. RMIN on the other hand was not significantly associated with any of the tested outcomes upon including covariates, apart from a statistical artifact in which the non-significant bivariate association between RMIN and meaning became significant in Sample 1.

In general, the results appear to very preliminarily suggest that the residualized approach to measuring mixed emotions may have relatively poorer predictive validity when used in models predicting key outcomes of interest. However, we urge very strong caution in the interpretation of these findings given that the use of residualized mixed emotion measures in predictive regression models are not as well-established in previous work. Moreover, as noted, previous usage of standardized residuals has primarily been in within-subjects approaches with multiple measurement occasions, and using it in a single-session cross-sectional context may not be appropriate, which could be one reason for these results.

**Appendix A—Materials**

**Emotion Scales**

To what extent did you feel the following emotions over the **past 15 minutes**?

**Sample 1 (Oh, 2020)**

Positive Emotions

1. Proud
2. Confident
3. Content
4. Satisfied
5. Grateful
6. Love
7. Admiration
8. Determined
9. Hopeful
10. Inspired
11. Courageous
12. Happy
13. Amused
14. Relieved
15. Excited
16. Relaxed
17. Interested
18. Awe
19. Impressed

Negative Emotions

1. Sad
2. Guilty
3. Ashamed
4. Regretful
5. Afraid
6. Fearful
7. Anxious
8. Embarrassed
9. Angry
10. Jealous
11. Boredom
12. Disgusted
13. Disappointed
14. Dependent
15. Hostile
16. Irritated
17. Contempt
18. Stressed

Mixed Emotions

1. A mix of both happiness and sadness at the same time
2. A mix of both anger and pride at the same time
3. A mix of both amusement and disgust at the same time
4. A mix of both regret and determination at the same time
5. A mix of both jealousy and admiration at the same time
6. A mix of both love and sadness at the same time
7. A mix of both hope and fear at the same time
8. A mix of both sadness and gratitude at the same time
9. A mix of both contentment and sadness at the same time
10. A mix of both determination and sadness at the same time
11. A mix of both relief and disappointment at the same time
12. A mix of both pride and fear at the same time
13. A mix of both gratitude and guilt at the same time
14. A mix of both regret and pride at the same time
15. A mix of both fear and courage at the same time
16. A mix of both sadness and amusement at the same time
17. A mix of both anger and determination at the same time
18. Bittersweet
19. Sentimental
20. Nostalgic
21. A combination of opposing emotions at the same time
22. A mixture of positive and negative emotions at the same time
23. Contrasting positive and negative emotions at the same time

**Sample 2**

Positive and Negative Emotions (PANAS; Watson et al., 1988)

1. Interested
2. Excited
3. Strong
4. Enthusiastic
5. Proud
6. Alert
7. Inspired
8. Determined
9. Attentive
10. Active
11. Distressed
12. Upset
13. Guilty
14. Scared
15. Hostile
16. Irritable
17. Ashamed
18. Nervous
19. Jittery
20. Afraid

Mixed Emotions (Barford & Smillie, 2016)

1. Both excited and nervous at the same time
2. Both amused and disgusted at the same time
3. Both bad and good at the same time
4. Both happy and sad at the same time
5. Both fearful and hopeful at the same time
6. Both positive and negative at the same time
7. Conflicted
8. Bittersweet
9. Nostalgic
10. Sentimental
11. A combination of positive and negative emotions at the same time
12. A mixture of positive and negative emotions at the same time
13. Contrasting positive and negative emotions at the same time

**Outcome Scales**

On the following pages, please answer the questions based on whether they describe how you are feeling **at this present moment**. That is to say, answer these questions based on whether they describe your **current** thoughts, feelings, or actions, rather than whether they might describe you in general. You do not need to dwell on any single item for too long - simply give your honest, instinctive response.

Psychological Conflict Scale

1. I feel torn between two opposing sides.
2. I feel a sense of internal conflict.
3. My present state consists of a great deal of contradiction.
4. My current thoughts feel highly contradictory.
5. Many conflicting things are going through my mind right now.
6. I do not feel any sense of contradiction. [R]
7. My current thoughts feel harmonious and unconflicted. [R]

Receptivity

1. I would be open to receiving both positive and negative feedback about myself.
2. I would be receptive to considering conflicting information before making a decision.
3. I would be willing to consider different perspectives even if they are conflicting.
4. I would be open to accepting new ways of seeing things.
5. I would be receptive to trying new behaviors.
6. I would not be keen on receiving conflicting information when making choices. [R]
7. I would not be keen on receiving information that challenges my perspective. [R]

Meaning in Life (Psychological Well-Being; Costin & Vignoles, 2020)

*Meaning*

1. My life right now has meaning.
2. My entire existence is full of meaning.
3. My life is meaningless. (R)
4. My existence is empty of meaning. (R)

*Coherence*

1. I can make sense of the things that happen in my life.
2. Looking at my life right now, things seem clear to me.
3. I can’t make sense of events in my life. (R)
4. My life feels like a sequence of unconnected events. (R)

*Purpose*

1. I have a good sense of what I am trying to accomplish in life.
2. I have certain life goals that compel me to keep going.
3. I don’t know what I am trying to accomplish in life. (R)
4. I don’t have compelling life goals that keep me going. (R)

*Mattering*

1. Whether my life ever existed matters even in the grand scheme of the universe.
2. Even considering how big the universe is, I can say that my life matters.
3. My existence is not significant in the grand scheme of things. (R)
4. Given the vastness of the universe, my life does not matter. (R)

**Controls**

On the following pages, please answer the questions based on whether they describe you **in general**. That is to say, answer these questions based on whether they describe your thoughts, feelings, or actions **most of the time**, even if they do not reflect you right now. You do not need to dwell on any single item for too long - simply give your honest, instinctive response.

Trait Dialecticism (Spencer-Rodgers et al., 2015)

1. When I hear two sides of an argument, I often agree with both.
2. I often find that things will contradict each other.
3. I sometimes believe two things that contradict each other.
4. My world is full of contradictions that cannot be resolved.
5. If there are two opposing sides to an argument, they cannot both be right. (reversed)
6. Believing two things that contradict each other is illogical. (reversed)
7. I find that if I look hard enough, I can figure out which side of a controversial issue is right. (reversed)
8. For most important issues, there is one right answer. (reversed)
9. I find that my world is relatively stable and consistent. (reversed)
10. When two sides disagree, the truth is always somewhere in the middle.
11. When I am solving a problem, I focus on finding the truth. (reversed)
12. When two of my friends disagree, I usually have a hard time deciding which of them is right.
13. There are always two sides to everything, depending on how you look at it.

Need for Cognition (Coelho et al., 2020)

1. I would prefer complex to simple problems.
2. I like to have the responsibility of handling a situation that requires a lot of thinking.
3. Thinking is not my idea of fun. (R)
4. I would rather do something that requires little thought than something that is sure to challenge my thinking abilities. (R)
5. I really enjoy a task that involves coming up with new solutions to problems.
6. I would prefer a task that is intellectual, difficult, and important to one that is somewhat important but does not require much thought.

Social Desirability (BIDR-16 Ver 6; Hart et al., 2015)

1. I have not always been honest with myself. (R)
2. I always know why I like things.
3. It’s hard for me to shut off a disturbing thought. (R)
4. I never regret my decisions.
5. I sometimes lose out on things because I can’t make my mind up soon enough.(R)
6. I am a completely rational person.
7. I am very confident of my judgement.
8. I have sometimes doubted my own ability as a lover. (R)
9. I sometimes tell lies if I have to. (R)
10. I never cover up my mistakes.
11. There have been occasions when I have taken advantage of someone. (R)
12. I sometimes try to get even rather than forgive and forget. (R)
13. I have said something bad about a friend behind his or her back. (R)
14. When I hear people talking privately, I avoid listening.
15. I never take things that don’t belong to me.
16. I don’t gossip about other people’s business.

Acquiescence Bias (Based on Kam, 2016)

1. I am fond of wooden furniture.
2. I like animals.
3. I enjoy salty food.
4. I am interested in electronics.
5. I have strong opinions about politics.
6. I am a good cook.
7. I am a morning person.
8. I like dark colors.
9. I dislike taking public transportation.
10. I am not interested in watching sports.
11. I do not like hot climates.
12. I cannot tolerate spicy food.
13. I do not enjoy superhero movies.
14. I am not skilled with using technology.
15. I am not fond of surprises.
16. I do not enjoy cold drinks.

Table S1. Correlation Matrix of all variables for Sample 1.

| Sample 1 Correlation Matrix | | | | | | | | | | | | | | | | | |
| --- | --- | --- | --- | --- | --- | --- | --- | --- | --- | --- | --- | --- | --- | --- | --- | --- | --- |
|  | 1 | 2 | 3 | 4 | 5 | 6 | 7 | 8 | 9 | 10 | 11 | 12 | 13 | 14 | 15 | 16 | 17 |
| 1. Age | - |  |  |  |  |  |  |  |  |  |  |  |  |  |  |  |  |
| 2. Gender | -.03 | - |  |  |  |  |  |  |  |  |  |  |  |  |  |  |  |
| 3. Education | -.004 | .08 | - |  |  |  |  |  |  |  |  |  |  |  |  |  |  |
| 4. Income | .04 | .12^*^ | .32^***^ | - |  |  |  |  |  |  |  |  |  |  |  |  |  |
| 5. Pos.E | .07 | .11^*^ | .07 | .16^***^ | - |  |  |  |  |  |  |  |  |  |  |  |  |
| 6. Neg.E | -.16^**^ | -.01 | .06 | -.09 | -.03 | - |  |  |  |  |  |  |  |  |  |  |  |
| 7. Mix.E (DM) | -.10 | .06 | .08 | -.04 | .28^***^ | .71^***^ | - |  |  |  |  |  |  |  |  |  |  |
| 8. MIN | -.13^**^ | .08 | .06 | .004 | .25^***^ | .80^***^ | .81^***^ | - |  |  |  |  |  |  |  |  |  |
| 9. Conflict | -.13^*^ | -.03 | .05 | -.11^*^ | -.10 | .59^***^ | .66^***^ | .55^***^ | - |  |  |  |  |  |  |  |  |
| 10. Receptivity | .05 | .03 | .09 | .09 | .29^***^ | -.16^**^ | -.01 | -.07 | -.10 | - |  |  |  |  |  |  |  |
| 11. Meaning | .19^***^ | .09 | .07 | .14^**^ | .44^***^ | -.51^***^ | -.23^***^ | -.28^***^ | -.42^***^ | .36^***^ | - |  |  |  |  |  |  |
| 12. Coherence | .13^*^ | .10 | .07 | .14^**^ | .37^***^ | -.52^***^ | -.36^***^ | -.35^***^ | -.53^***^ | .31^***^ | .78^***^ | - |  |  |  |  |  |
| 13. Purpose | .17^***^ | .05 | .04 | .13^*^ | .34^***^ | -.44^***^ | -.30^***^ | -.33^***^ | -.47^***^ | .35^***^ | .83^***^ | .79^***^ | - |  |  |  |  |
| 14. Mattering | .19^***^ | .05 | .05 | .11^*^ | .47^***^ | -.32^***^ | -.08 | -.12^*^ | -.29^***^ | .34^***^ | .77^***^ | .59^***^ | .62^***^ | - |  |  |  |
| 15. Trait Dia. | -.03 | -.15^**^ | .05 | -.04 | -.09 | .24^***^ | .33^***^ | .24^***^ | .43^***^ | .30^***^ | -.22^***^ | -.29^***^ | -.24^***^ | -0.13^*^ | - |  |  |
| 16. NFC | .13^*^ | .13^*^ | .23^***^ | .15^**^ | .27^***^ | -.16^**^ | -.02 | -.06 | -.10 | .43^***^ | .29^***^ | .24^***^ | .26^***^ | .21^***^ | .02 | - |  |
| 17. SDS | .13^*^ | .11^*^ | .03 | .11^*^ | .35^***^ | -.41^***^ | -.28^***^ | -.29^***^ | -.41^***^ | .19^***^ | .52^***^ | .56^***^ | .48^***^ | .43^***^ | -.34^***^ | .26^***^ | - |
| 18. ACQ | .09 | -.22^***^ | -.12^*^ | -.08 | -.02 | .06 | 0.04 | .07 | .02 | -.10^*^ | -.08^*^ | -.06 | -.02 | -.11^*^ | .01 | -.05 | -0.04 |

^*^ *p* < .05, ^**^ *p* < .01, ^***^ *p* < .001. Pos E = Positive Emotions; Neg E = Negative Emotions; Mix E = Mixed Emotions (Direct Measure); MIN = Minimum Index; Trait Dia. = Trait Dialecticism; NFC = Need for Cognition; SDS = Social Desirability; ACQ = Acquiescence

Table S2. Correlation Matrix of all variables for Sample 2.

| Sample 2 Correlation Matrix | | | | | | | | | | | | | | | | | |
| --- | --- | --- | --- | --- | --- | --- | --- | --- | --- | --- | --- | --- | --- | --- | --- | --- | --- |
|  | 1 | 2 | 3 | 4 | 5 | 6 | 7 | 8 | 9 | 10 | 11 | 12 | 13 | 14 | 15 | 16 | 17 |
| 1. Age | - |  |  |  |  |  |  |  |  |  |  |  |  |  |  |  |  |
| 2. Gender | -.11^*^ | - |  |  |  |  |  |  |  |  |  |  |  |  |  |  |  |
| 3. Education | .05 | .05 | - |  |  |  |  |  |  |  |  |  |  |  |  |  |  |
| 4. Income | -.03 | .06 | .33^***^ | - |  |  |  |  |  |  |  |  |  |  |  |  |  |
| 5. Pos E | .13^*^ | .09 | .06 | .19^***^ | - |  |  |  |  |  |  |  |  |  |  |  |  |
| 6. Neg E | -.16^**^ | -.02 | -.07 | -.11^*^ | -.07 | - |  |  |  |  |  |  |  |  |  |  |  |
| 7. Mix E (DM) | -.09 | -.07 | -.01 | -.05 | .17^***^ | .60^***^ | - |  |  |  |  |  |  |  |  |  |  |
| 8. MIN | -.17^***^ | 0 | -.05 | -.10^*^ | .03 | .94^***^ | .66^***^ | - |  |  |  |  |  |  |  |  |  |
| 9. Conflict | -.08 | -.08 | -.11^*^ | -.13^*^ | -.12^*^ | .57^***^ | .66^***^ | .57^***^ | - |  |  |  |  |  |  |  |  |
| 10. Receptivity | -.02 | .04 | .04 | .09 | .23^***^ | -.08 | .01 | -.06 | -.06 | - |  |  |  |  |  |  |  |
| 11. Meaning | .11^*^ | -.07 | .10^*^ | .24^***^ | .46^***^ | -.29^***^ | -.12^*^ | -.23^***^ | -.28^***^ | .22^***^ | - |  |  |  |  |  |  |
| 12. Coherence | .12^*^ | 0 | .14^**^ | .27^***^ | .41^***^ | -.36^***^ | -.25^***^ | -.30^***^ | -.43^***^ | .22^***^ | .73^***^ | - |  |  |  |  |  |
| 13. Purpose | .10 | .01 | .12^*^ | .29^***^ | .46^***^ | -.31^***^ | -.19^***^ | -.25^***^ | -.37^***^ | .25^***^ | .82^***^ | .75^***^ | - |  |  |  |  |
| 14. Mattering | .19^***^ | -.06 | .08 | .16^**^ | .43^***^ | -.24^***^ | -.12^*^ | -.21^***^ | -.26^***^ | .13^*^ | .82^***^ | .59^***^ | .70^***^ | - |  |  |  |
| 15. Trait Dia. | -.08 | -.16^**^ | .02 | -.14^**^ | -.12^*^ | .27^***^ | .32^***^ | .26^***^ | .39^***^ | .18^***^ | -.25^***^ | -.29^***^ | -.33^***^ | -.20^***^ | - |  |  |
| 16. NFC | .07 | .13^*^ | .13^**^ | .19^***^ | .30^***^ | -.10 | .07 | -.05 | -.08 | .44^***^ | .28^***^ | .27^***^ | .30^***^ | .26^***^ | -.04 | - |  |
| 17. SDS | .12^*^ | .04 | .03 | .16^**^ | .32^***^ | -.36^***^ | -.25^***^ | -.32^***^ | -.39^***^ | .06 | .39^***^ | .43^***^ | .42^***^ | .38^***^ | -.32^***^ | .23^***^ | - |
| 18. ACQ | .11^*^ | -.24^***^ | -.05 | -.17^**^ | .08 | .15^**^ | .24^***^ | .18^***^ | .16^**^ | -.03 | -.14^**^ | -.07 | -.17^**^ | -.15^**^ | .07 | -.11^*^ | -.08 |

^*^ *p* < .05, ^**^ *p* < .01, ^***^ *p* < .001. Pos E = Positive Emotions; Neg E = Negative Emotions; Mix E = Mixed Emotions (Direct Measure); MIN = Minimum Index; Trait Dia. = Trait Dialecticism; NFC = Need for Cognition; SDS = Social Desirability; ACQ = Acquiescence

Table S3. Models using direct measures, controlling for all covariates (Sample 1).

|  | Conflict | | | | |  | Receptivity | | | | |
| --- | --- | --- | --- | --- | --- | --- | --- | --- | --- | --- | --- |
|  | *b* | *SE* | *p* | *β* | 95% CI |  | *b* | *SE* | *p* | *β* | 95% CI |
| Mixed Emotions | 0.71^***^ | 0.08 | <.001 | .55 | [0.56, 0.86] |  | -0.15^*^ | 0.08 | .045 | -0.14 | [-0.30, -0.003] |
| Positive Emotions | -0.21^***^ | 0.05 | <.001 | -.20 | [-0.30, -0.12] |  | 0.23^***^ | 0.05 | <.001 | 0.26 | [0.14, 0.32] |
| Negative Emotions | 0.16^*^ | 0.08 | .036 | .12 | [0.01, 0.30] |  | -0.07 | 0.08 | .36 | -0.06 | [-0.22, 0.08] |
| Age | -0.002 | 0.004 | .56 | -.02 | [-0.01, 0.01] |  | -0.002 | 0.004 | .55 | -0.03 | [-0.01, 0.01] |
| Gender | -0.01 | 0.11 | .91 | -.004 | [-0.23, 0.21] |  | 0.02 | 0.11 | .87 | 0.01 | [-0.20, 0.24] |
| Education Level | 0.02 | 0.03 | .57 | .02 | [-0.04, 0.07] |  | -0.01 | 0.03 | .82 | -0.01 | [-0.06, 0.05] |
| Income | -0.02 | 0.02 | .40 | -.03 | [-0.05, 0.02] |  | -0.002 | 0.02 | .92 | -0.01 | [-0.04, 0.04] |
| Trait Dialecticism | 0.30^***^ | 0.07 | <.001 | .17 | [0.16, 0.44] |  | 0.63^***^ | 0.07 | <.001 | 0.42 | [0.49, 0.77] |
| Need for Cognition | -0.01 | 0.03 | .77 | -.01 | [-0.08, 0.06] |  | 0.22^***^ | 0.03 | <.001 | 0.31 | [0.16, 0.29] |
| Social Desirability | -0.09 | 0.06 | .12 | -.07 | [-0.20, 0.02] |  | 0.12^*^ | 0.06 | .040 | 0.11 | [0.01, 0.23] |
| Acquiescence | -0.02 | 0.10 | .81 | -.01 | [-0.22, 0.17] |  | -0.17 | 0.10 | .094 | -0.07 | [-0.36, 0.03] |
|  | Meaning | | | | |  | Coherence | | | | |
|  | *b* | *SE* | *p* | *β* | 95% CI |  | *b* | *SE* | *p* | *β* | 95% CI |
| Mixed Emotions | 0.09 | 0.09 | .31 | .06 | [-0.08, 0.26] |  | -0.21^**^ | 0.07 | .005 | -.18 | [-0.35, -0.06] |
| Positive Emotions | 0.36^***^ | 0.05 | <.001 | .32 | [0.26, 0.46] |  | 0.28^***^ | 0.05 | <.001 | .30 | [0.19, 0.37] |
| Negative Emotions | -0.64^***^ | 0.09 | <.001 | -.44 | [-0.81, -0.48] |  | -0.31^***^ | 0.07 | <.001 | -.26 | [-0.46, -0.17] |
| Age | 0.01 | 0.01 | .12 | .06 | [-0.002, 0.02] |  | 0.001 | 0.004 | .89 | .01 | [-0.01, 0.01] |
| Gender | -0.002 | 0.13 | .99 | -.001 | [-0.25, 0.25] |  | 0.09 | 0.11 | .39 | .04 | [-0.12, 0.31] |
| Education Level | 0.03 | 0.03 | .37 | .04 | [-0.03, 0.09] |  | 0.04 | 0.03 | .19 | .06 | [-0.02, 0.09] |
| Income | 0.001 | 0.02 | .96 | .002 | [-0.04, 0.04] |  | 0.002 | 0.02 | .91 | .01 | [-0.03, 0.04] |
| Trait Dialecticism | -0.05 | 0.08 | .52 | -.03 | [-0.21, 0.11] |  | -0.07 | 0.07 | .29 | -.05 | [-0.21, 0.06] |
| Need for Cognition | 0.07 | 0.04 | .083 | .07 | [-0.01, 0.14] |  | 0.03 | 0.03 | .33 | .04 | [-0.03, 0.10] |
| Social Desirability | 0.26^***^ | 0.07 | <.001 | .19 | [0.13, 0.39] |  | 0.30^***^ | 0.06 | <.001 | .26 | [0.19, 0.41] |
| Acquiescence | -0.15 | 0.11 | .18 | -.05 | [-0.38, 0.07] |  | -0.01 | 0.10 | .88 | -.01 | [-0.21, 0.18] |
|  | Purpose | | | | |  | Mattering | | | | |
|  | *b* | *SE* | *p* | *β* | 95% CI |  | *b* | *SE* | *p* | *β* | 95% CI |
| Mixed Emotions | -0.16 | 0.09 | .088 | -.12 | [-0.34, 0.02] |  | 0.14 | 0.11 | .19 | .09 | [-0.07, 0.36] |
| Positive Emotions | 0.28^***^ | 0.06 | <.001 | .26 | [0.17, 0.39] |  | 0.46^***^ | 0.07 | <.001 | .37 | [0.33, 0.59] |
| Negative Emotions | -0.32^***^ | 0.09 | <.001 | -.23 | [-0.50, -0.14] |  | -0.46^***^ | 0.11 | <.001 | -.28 | [-0.67, -0.25] |
| Age | 0.01 | 0.01 | .17 | .06 | [-0.003, 0.02] |  | 0.02^*^ | 0.01 | .011 | .11 | [0.003, 0.03] |
| Gender | -0.06 | 0.14 | .64 | -.02 | [-0.33, 0.20] |  | -0.11 | 0.16 | .50 | -.03 | [-0.42, 0.21] |
| Education Level | 0.02 | 0.03 | .64 | .02 | [-0.05, 0.08] |  | 0.02 | 0.04 | .63 | .02 | [-0.06, 0.10] |
| Income | 0.01 | 0.02 | .74 | .02 | [-0.04, 0.05] |  | -0.01 | 0.03 | .73 | -.02 | [-0.06, 0.04] |
| Trait Dialecticism | -0.09 | 0.09 | .28 | -.05 | [-0.26, 0.08] |  | 0.03 | 0.10 | .79 | .01 | [-0.17, 0.23] |
| Need for Cognition | 0.08 | 0.04 | .068 | .09 | [-0.01, 0.16] |  | 0.01 | 0.05 | .93 | .004 | [-0.09, 0.10] |
| Social Desirability | 0.26^***^ | 0.07 | <.001 | .20 | [0.12, 0.39] |  | 0.29^***^ | 0.08 | <.001 | .19 | [0.13, 0.45] |
| Acquiescence | 0.01 | 0.12 | .91 | .01 | [-0.22, 0.25] |  | -0.32^*^ | 0.14 | .023 | -.10 | [-0.61, -0.04] |

^*^ *p* < .05, ^**^ *p* < .01, ^***^ *p* < .001. Gender was coded with 1 = *Males* and 0 = *Females.*

Table S4. Models using MIN, controlling for all covariates (Sample 1).

|  | Conflict | | | | |  | Receptivity | | | | |
| --- | --- | --- | --- | --- | --- | --- | --- | --- | --- | --- | --- |
|  | *b* | *SE* | *p* | *β* | 95% CI |  | *b* | *SE* | *p* | *β* | 95% CI |
| MIN | 0.51^***^ | 0.13 | <.001 | 0.30 | [0.26, 0.76] |  | -0.16 | 0.12 | .17 | -0.11 | [-0.39, 0.07] |
| Positive Emotions | -0.10 | 0.05 | .055 | -0.09 | [-0.19, 0.002] |  | 0.22^***^ | 0.05 | <.001 | 0.25 | [0.13, 0.31] |
| Negative Emotions | 0.33^**^ | 0.10 | .001 | 0.25 | [0.14, 0.52] |  | -0.07 | 0.09 | .41 | -0.07 | [-0.25, 0.10] |
| Age | -0.002 | 0.004 | .64 | -0.02 | [-0.01, 0.01] |  | -0.003 | 0.004 | .52 | -0.03 | [-0.01, 0.01] |
| Gender | 0.05 | 0.12 | .71 | 0.02 | [-0.19, 0.29] |  | 0.01 | 0.11 | .90 | 0.01 | [-0.21, 0.23] |
| Education Level | 0.02 | 0.03 | .45 | 0.03 | [-0.04, 0.08] |  | -0.01 | 0.03 | .76 | -0.01 | [-0.06, 0.05] |
| Income | -0.03 | 0.02 | .15 | -0.06 | [-0.07, 0.01] |  | 0.002 | 0.02 | .93 | 0.004 | [-0.04, 0.04] |
| Trait Dialecticism | 0.44^***^ | 0.08 | <.001 | 0.25 | [0.30, 0.59] |  | 0.60^***^ | 0.07 | <.001 | 0.40 | [0.47, 0.74] |
| Need for Cognition | -0.01 | 0.04 | .86 | -0.01 | [-0.08, 0.07] |  | 0.22^***^ | 0.03 | <.001 | 0.31 | [0.16, 0.29] |
| Social Desirability | -0.12 | 0.06 | .058 | -0.09 | [-0.24, 0.004] |  | 0.12^*^ | 0.06 | .035 | 0.11 | [0.01, 0.23] |
| Acquiescence | -0.03 | 0.11 | .82 | -0.01 | [-0.24, 0.19] |  | -0.16 | 0.10 | .11 | -0.07 | [-0.36, 0.03] |
|  | Meaning | | | | |  | Coherence | | | | |
|  | *b* | *SE* | *p* | *β* | 95% CI |  | *b* | *SE* | *p* | *β* | 95% CI |
| MIN | 0.16 | 0.13 | .23 | .09 | [-0.10, 0.42] |  | -0.11 | 0.12 | .33 | -.07 | [-0.34, 0.11] |
| Positive Emotions | 0.36^***^ | 0.05 | <.001 | .32 | [0.25, 0.46] |  | 0.24^***^ | 0.05 | <.001 | .26 | [0.15, 0.33] |
| Negative Emotions | -0.68^***^ | 0.10 | <.001 | -.47 | [-0.88, -0.48] |  | -0.39^***^ | 0.09 | <.001 | -.32 | [-0.56, -0.22] |
| Age | 0.01 | 0.01 | .11 | .06 | [-0.002, 0.02] |  | 0.001 | 0.004 | .90 | .01 | [-0.01, 0.01] |
| Gender | -0.01 | 0.13 | .95 | -.003 | [-0.26, 0.24] |  | 0.07 | 0.11 | .52 | .03 | [-0.15, 0.29] |
| Education Level | 0.03 | 0.03 | .33 | .04 | [-0.03, 0.09] |  | 0.03 | 0.03 | .22 | .05 | [-0.02, 0.09] |
| Income | -0.002 | 0.02 | .93 | -.004 | [-0.04, 0.04] |  | 0.01 | 0.02 | .76 | .01 | [-0.03, 0.04] |
| Trait Dialecticism | -0.04 | 0.08 | .60 | -.02 | [-0.20, 0.11] |  | -0.12 | 0.07 | .079 | -.08 | [-0.25, 0.01] |
| Need for Cognition | 0.07 | 0.04 | .084 | .07 | [-0.01, 0.14] |  | 0.03 | 0.03 | .36 | .04 | [-0.04, 0.10] |
| Social Desirability | 0.26^***^ | 0.06 | <.001 | .19 | [0.13, 0.39] |  | 0.31^***^ | 0.06 | <.001 | .27 | [0.20, 0.42] |
| Acquiescence | -0.16 | 0.11 | .16 | -.06 | [-0.39, 0.06] |  | -0.02 | 0.10 | .86 | -.01 | [-0.21, 0.18] |
|  | Purpose | | | | |  | Mattering | | | | |
|  | *b* | *SE* | *p* | *β* | 95% CI |  | *b* | *SE* | *p* | *β* | 95% CI |
| MIN | -0.31^*^ | 0.14 | .027 | -.18 | [-0.59, -0.04] |  | 0.18 | 0.17 | .27 | .09 | [-0.14, 0.51] |
| Positive Emotions | 0.29^***^ | 0.06 | <.001 | .27 | [0.18, 0.40] |  | 0.47^***^ | 0.07 | <.001 | .37 | [0.34, 0.60] |
| Negative Emotions | -0.24^*^ | 0.11 | .026 | -.17 | [-0.45, -0.03] |  | -0.47^***^ | 0.13 | <.001 | -.29 | [-0.72, -0.22] |
| Age | 0.01 | 0.01 | .21 | .05 | [-0.003, 0.02] |  | 0.02^*^ | 0.01 | .010 | .11 | [0.004, 0.03] |
| Gender | -0.05 | 0.14 | .73 | -.02 | [-0.31, 0.22] |  | -0.11 | 0.16 | .51 | -.03 | [-0.42, 0.21] |
| Education Level | 0.01 | 0.03 | .72 | .02 | [-0.05, 0.08] |  | 0.02 | 0.04 | .59 | .03 | [-0.06, 0.10] |
| Income | 0.01 | 0.02 | .57 | .03 | [-0.03, 0.06] |  | -0.01 | 0.03 | .63 | -.02 | [-0.07, 0.04] |
| Trait Dialecticism | -0.11 | 0.08 | .19 | -.06 | [-0.27, 0.05] |  | 0.05 | 0.10 | .61 | .02 | [-0.14, 0.25] |
| Need for Cognition | 0.08 | 0.04 | .065 | .09 | [-0.01, 0.16] |  | 0.01 | 0.05 | .92 | .01 | [-0.09, 0.10] |
| Social Desirability | 0.25^***^ | 0.07 | <.001 | .20 | [0.12, 0.39] |  | 0.29^***^ | 0.08 | <.001 | .19 | [0.13, 0.45] |
| Acquiescence | 0.03 | 0.12 | .81 | .01 | [-0.21, 0.27] |  | -0.33^*^ | 0.14 | .021 | -.10 | [-0.61, -0.05] |

^*^ *p* < .05, ^**^ *p* < .01, ^***^ *p* < .001. Gender was coded with 1 = *Males* and 0 = *Females.*

Table S5. Models using direct measures, controlling for all covariates (Sample 2).

|  | Conflict | | | | |  | Receptivity | | | | |
| --- | --- | --- | --- | --- | --- | --- | --- | --- | --- | --- | --- |
|  | *b* | *SE* | *p* | *β* | 95% CI |  | *b* | *SE* | *p* | *β* | 95% CI |
| Mixed Emotions | 0.64^***^ | 0.06 | <.001 | .52 | [0.52, 0.75] |  | -0.12^*^ | 0.06 | .034 | -.13 | [-0.23, -0.01] |
| Positive Emotions | -0.17^***^ | 0.04 | <.001 | -.16 | [-0.25, -0.08] |  | 0.14^**^ | 0.04 | .001 | .18 | [0.06, 0.22] |
| Negative Emotions | 0.26^***^ | 0.07 | <.001 | .18 | [0.13, 0.40] |  | -0.06 | 0.07 | .36 | -.05 | [-0.19, 0.07] |
| Age | 0.01 | 0.004 | .16 | .05 | [-0.002, 0.01] |  | -0.01 | 0.004 | .16 | -.06 | [-0.01, 0.002] |
| Gender | 0.04 | 0.11 | .74 | .01 | [-0.18, 0.25] |  | -0.002 | 0.11 | .99 | -.001 | [-0.21, 0.21] |
| Education Level | -0.06^*^ | 0.03 | .029 | -.08 | [-0.11, -0.01] |  | -0.02 | 0.03 | .37 | -.04 | [-0.07, 0.03] |
| Income | 0.01 | 0.02 | .74 | .01 | [-0.03, 0.04] |  | 0.01 | 0.02 | .70 | .02 | [-0.03, 0.04] |
| Trait Dialecticism | 0.24^***^ | 0.07 | <.001 | .14 | [0.11, 0.37] |  | 0.32^***^ | 0.06 | <.001 | .25 | [0.20, 0.45] |
| Need for Cognition | -0.02 | 0.03 | .54 | -.02 | [-0.09, 0.05] |  | 0.29^***^ | 0.03 | <.001 | .43 | [0.22, 0.35] |
| Social Desirability | -0.13^*^ | 0.05 | .015 | -.10 | [-0.24, -0.03] |  | -0.04 | 0.05 | .40 | -.04 | [-0.15, 0.06] |
| Acquiescence | -0.004 | 0.10 | .97 | -.001 | [-0.21, 0.20] |  | 0.03 | 0.10 | .76 | .01 | [-0.16, 0.22] |
|  | Meaning | | | | |  | Coherence | | | | |
|  | *b* | *SE* | *p* | *β* | 95% CI |  | *b* | *SE* | *p* | *β* | 95% CI |
| Mixed Emotions | -0.03 | 0.07 | .70 | -.02 | [-0.17, 0.12] |  | -0.17^**^ | 0.06 | .005 | -.16 | [-0.29, -0.05] |
| Positive Emotions | 0.44^***^ | 0.06 | <.001 | .38 | [0.33, 0.54] |  | 0.29^***^ | 0.05 | <.001 | .31 | [0.20, 0.38] |
| Negative Emotions | -0.20^*^ | 0.09 | .020 | -.13 | [-0.37, -0.03] |  | -0.15^*^ | 0.07 | .033 | -.12 | [-0.29, -0.01] |
| Age | -0.001 | 0.01 | .89 | -.01 | [-0.01, 0.01] |  | 0.001 | 0.004 | .87 | .01 | [-0.01, 0.01] |
| Gender | -0.62^***^ | 0.14 | <.001 | -.19 | [-0.90, -0.35] |  | -0.24^*^ | 0.12 | .036 | -.09 | [-0.47, -0.02] |
| Education Level | 0.03 | 0.03 | .46 | .03 | [-0.04, 0.09] |  | 0.04 | 0.03 | .21 | .06 | [-0.02, 0.09] |
| Income | 0.04 | 0.02 | .11 | .07 | [-0.01, 0.08] |  | 0.05^*^ | 0.02 | .010 | .12 | [0.01, 0.08] |
| Trait Dialecticism | -0.24^**^ | 0.09 | .004 | -.13 | [-0.41, -0.08] |  | -0.18^*^ | 0.07 | .011 | -.12 | [-0.31, -0.04] |
| Need for Cognition | 0.11^**^ | 0.04 | .008 | .12 | [0.03, 0.20] |  | 0.09^**^ | 0.04 | .008 | .12 | [0.03, 0.16] |
| Social Desirability | 0.18^**^ | 0.07 | .009 | .13 | [0.05, 0.32] |  | 0.19^**^ | 0.06 | .001 | .16 | [0.08, 0.30] |
| Acquiescence | -0.43^**^ | 0.13 | .001 | -.15 | [-0.69, -0.17] |  | -0.01 | 0.11 | .93 | -.004 | [-0.22, 0.20] |
|  | Purpose | | | | |  | Mattering | | | | |
|  | *b* | *SE* | *p* | *β* | 95% CI |  | *b* | *SE* | *p* | *β* | 95% CI |
| Mixed Emotions | -0.11 | 0.06 | .081 | -.09 | [-0.24, 0.01] |  | -0.06 | 0.08 | .46 | -.04 | [-0.23, 0.10] |
| Positive Emotions | 0.39^***^ | 0.05 | <.001 | .37 | [0.29, 0.48] |  | 0.46^***^ | 0.06 | <.001 | .36 | [0.33, 0.58] |
| Negative Emotions | -0.12 | 0.08 | .11 | -.08 | [-0.27, 0.03] |  | -0.12 | 0.10 | .24 | -.07 | [-0.31, 0.08] |
| Age | 0.00 | 0.00 | .77 | -.01 | [-0.01, 0.01] |  | 0.01 | 0.01 | .057 | .08 | [-\|<0.001\|, 0.02] |
| Gender | -0.38^**^ | 0.12 | .002 | -.13 | [-0.62, -0.14] |  | -0.63^***^ | 0.16 | <.001 | -.17 | [-0.94, -0.31] |
| Education Level | 0.03 | 0.03 | .41 | .04 | [-0.03, 0.08] |  | 0.03 | 0.04 | .45 | .03 | [-0.05, 0.11] |
| Income | 0.05^*^ | 0.02 | .010 | .11 | [0.01, 0.09] |  | 0.01 | 0.03 | .80 | .01 | [-0.04, 0.06] |
| Trait Dialecticism | -0.32^***^ | 0.07 | <.001 | -.19 | [-0.47, -0.18] |  | -0.20^*^ | 0.10 | .039 | -.10 | [-0.39, -0.01] |
| Need for Cognition | 0.12^**^ | 0.04 | .002 | .13 | [0.04, 0.19] |  | 0.12^*^ | 0.05 | .012 | .12 | [0.03, 0.22] |
| Social Desirability | 0.17^**^ | 0.06 | .006 | .13 | [0.05, 0.28] |  | 0.23^**^ | 0.08 | .004 | .15 | [0.08, 0.39] |
| Acquiescence | -0.37^**^ | 0.12 | .001 | -.14 | [-0.59, -0.14] |  | -0.56^***^ | 0.15 | <.001 | -.17 | [-0.85, -0.26] |

^*^ *p* < .05, ^**^ *p* < .01, ^***^ *p* < .001. Gender was coded with 1 = *Males* and 0 = *Females.*

Table S6. Models using MIN, controlling for all covariates (Sample 2).

|  | Conflict | | | | |  | Receptivity | | | | |
| --- | --- | --- | --- | --- | --- | --- | --- | --- | --- | --- | --- |
|  | *b* | *SE* | *p* | *β* | 95% CI |  | *b* | *SE* | *p* | *β* | 95% CI |
| MIN | 0.63^**^ | 0.21 | .003 | .37 | [0.22, 1.03] |  | -0.15 | 0.18 | .38 | -.12 | [-0.50, 0.19] |
| Positive Emotions | -0.08 | 0.05 | .10 | -.08 | [-0.18, 0.02] |  | 0.13^**^ | 0.04 | .002 | .16 | [0.05, 0.21] |
| Negative Emotions | 0.16 | 0.18 | .38 | .11 | [-0.20, 0.51] |  | -0.01 | 0.15 | .95 | -.01 | [-0.31, 0.29] |
| Age | 0.01 | 0.01 | .25 | .05 | [-0.004, 0.01] |  | -0.01 | 0.004 | .16 | -.06 | [-0.01, 0.002] |
| Gender | -0.04 | 0.13 | .73 | -.02 | [-0.29, 0.20] |  | 0.01 | 0.11 | .90 | .01 | [-0.19, 0.22] |
| Education Level | -0.06^*^ | 0.03 | .043 | -.09 | [-0.12, -0.002] |  | -0.02 | 0.03 | .39 | -.04 | [-0.07, 0.03] |
| Income | 0.01 | 0.02 | .59 | .02 | [-0.03, 0.05] |  | 0.01 | 0.02 | .75 | .02 | [-0.03, 0.04] |
| Trait Dialecticism | 0.37^***^ | 0.07 | <.001 | .22 | [0.23, 0.52] |  | 0.30^***^ | 0.06 | <.001 | .23 | [0.18, 0.42] |
| Need for Cognition | 0.02 | 0.04 | .58 | .02 | [-0.05, 0.10] |  | 0.28^***^ | 0.03 | <.001 | .42 | [0.22, 0.34] |
| Social Desirability | -0.19^**^ | 0.06 | .002 | -.14 | [-0.31, -0.07] |  | -0.03 | 0.05 | .52 | -.03 | [-0.14, 0.07] |
| Acquiescence | 0.13 | 0.12 | .26 | .05 | [-0.10, 0.36] |  | 0.01 | 0.10 | .94 | .003 | [-0.19, 0.20] |
|  | Meaning | | | | |  | Coherence | | | | |
|  | *b* | *SE* | *P* | *β* | 95% CI |  | *b* | *SE* | *p* | *β* | 95% CI |
| MIN | 0.16 | 0.23 | .48 | .09 | [-0.29, 0.62] |  | 0.01 | 0.19 | .95 | .01 | [-0.37, 0.39] |
| Positive Emotions | 0.42^***^ | 0.06 | <.001 | .36 | [0.31, 0.53] |  | 0.25^***^ | 0.05 | <.001 | .27 | [0.17, 0.34] |
| Negative Emotions | -0.35 | 0.20 | .083 | -.22 | [-0.75, 0.05] |  | -0.27 | 0.17 | .11 | -.20 | [-0.60, 0.06] |
| Age | -\|<0.001\| | 0.01 | .96 | -.002 | [-0.01, 0.01] |  | 0.001 | 0.004 | .78 | .01 | [-0.01, 0.01] |
| Gender | -0.62^***^ | 0.14 | <.001 | -.19 | [-0.90, -0.35] |  | -0.23 | 0.12 | .053 | -.09 | [-0.46, 0.003] |
| Education Level | 0.02 | 0.03 | .48 | .03 | [-0.04, 0.09] |  | 0.04 | 0.03 | .22 | .06 | [-0.02, 0.09] |
| Income | 0.04 | 0.02 | .10 | .07 | [-0.01, 0.08] |  | 0.05^*^ | 0.02 | .011 | .12 | [0.01, 0.08] |
| Trait Dialecticism | -0.25^**^ | 0.08 | .002 | -.14 | [-0.42, -0.09] |  | -0.22^**^ | 0.07 | .002 | -.14 | [-0.35, -0.08] |
| Need for Cognition | 0.11^**^ | 0.04 | .009 | .12 | [0.03, 0.19] |  | 0.08^*^ | 0.04 | .021 | .10 | [0.01, 0.15] |
| Social Desirability | 0.19^**^ | 0.07 | .007 | .13 | [0.05, 0.32] |  | 0.20^***^ | 0.06 | <.001 | .17 | [0.09, 0.32] |
| Acquiescence | -0.45^**^ | 0.13 | .001 | -.15 | [-0.70, -0.19] |  | -0.06 | 0.11 | .61 | -.02 | [-0.27, 0.16] |
|  | Purpose | | | | |  | Mattering | | | | |
|  | *b* | *SE* | *p* | *β* | 95% CI |  | *b* | *SE* | *p* | *β* | 95% CI |
| MIN | 0.04 | 0.21 | .85 | .02 | [-0.36, 0.44] |  | -0.15 | 0.27 | .57 | -.07 | [-0.68, 0.37] |
| Positive Emotions | 0.36^***^ | 0.05 | <.001 | .35 | [0.27, 0.46] |  | 0.46^***^ | 0.06 | <.001 | .36 | [0.33, 0.58] |
| Negative Emotions | -0.22 | 0.18 | .21 | -.15 | [-0.57, 0.13] |  | -0.03 | 0.23 | .89 | -.02 | [-0.49, 0.42] |
| Age | -0.001 | 0.004 | .85 | -.01 | [-0.01, 0.01] |  | 0.01 | 0.01 | .063 | .08 | [-0.001, 0.02] |
| Gender | -0.37^**^ | 0.12 | .002 | -.13 | [-0.62, -0.13] |  | -0.62^***^ | 0.16 | <.001 | -.17 | [-0.93, -0.30] |
| Education Level | 0.02 | 0.03 | .42 | .03 | [-0.03, 0.08] |  | 0.03 | 0.04 | .44 | .04 | [-0.05, 0.11] |
| Income | 0.05^*^ | 0.02 | .010 | .11 | [0.01, 0.09] |  | 0.01 | 0.03 | .83 | .01 | [-0.04, 0.05] |
| Trait Dialecticism | -0.35^***^ | 0.07 | <.001 | -.21 | [-0.49, -0.21] |  | -0.21^*^ | 0.10 | .025 | -.10 | [-0.40, -0.03] |
| Need for Cognition | 0.11^**^ | 0.04 | .004 | .12 | [0.04, 0.18] |  | 0.12^*^ | 0.05 | .014 | .11 | [0.02, 0.22] |
| Social Desirability | 0.18^**^ | 0.06 | .004 | .14 | [0.06, 0.30] |  | 0.24^**^ | 0.08 | .003 | .15 | [0.08, 0.39] |
| Acquiescence | -0.40^**^ | 0.11 | .001 | -.15 | [-0.62, -0.17] |  | -0.57^***^ | 0.15 | <.001 | -.17 | [-0.86, -0.27] |

^*^ *p* < .05, ^**^ *p* < .01, ^***^ *p* < .001. Gender was coded with 1 = *Males* and 0 = *Females.*
